# Integrated Transcriptomic Analysis Reveals the Molecular Mechanism of Meningiomas by Weighted Gene Coexpression Network Analysis

**DOI:** 10.1155/2020/4927547

**Published:** 2020-06-10

**Authors:** Biao Yang, Shuxun Wei, Yan-Bin Ma, Sheng-Hua Chu

**Affiliations:** ^1^Department of Neurosurgery, Shanghai Ninth People's Hospital Affiliated to Shanghai Jiao Tong University School of Medicine, Shanghai 201999, China; ^2^Department of General Surgery, The Second Military Medical University/Changzheng Hospital, Shanghai 201999, China

## Abstract

Meningiomas are the most common primary intracranial tumor in adults. However, to date, systemic coexpression analyses for meningiomas fail to explain its pathogenesis. The aim of the present study was to construct coexpression modules and identify potential biomarkers associated with meningioma progression. Weighted gene coexpression network analysis (WGCNA) was performed based on GSE43290, and module preservation was tested by GSE74385. Functional annotations were performed to analyze biological significance. Hub genes were selected for efficacy evaluations and correlation analyses using two independent cohorts. A total of 14 coexpression modules were identified, and module lightcyan was significantly associated with WHO grades. Functional enrichment analyses of module lightcyan were associated with tumor pathogenesis. The top 10 hub genes were extracted. Ten biomarkers, particularly AHCYL2, FGL2, and KCNMA1, were significantly related to grades and prognosis of meningioma. These findings not only construct coexpression modules leading to the better understanding of its pathogenesis but also provide potential biomarkers that represent specific on tumor grades and identify recurrence, predicting prognosis and progression of meningiomas.

## 1. Introduction

Meningiomas are the most common primary intracranial tumor in adults, accounting for over 35% of intracranial tumors [1]. According to the 2007 World Health Organization (WHO) Classification of Tumors of the Central Nervous System (CNS), meningiomas are classified into three grades including grade I, grade II, and grade III [2]. In the updated 2016 classification, brain invasion was added into the diagnostic criteria of atypical meningioma, WHO grade II [3]. Approximately 80% of all cases are WHO grade I meningiomas, while the high-grade meningiomas, including WHO grade II or III, comprise 18-20% and 1-2% of all cases, respectively [4]. Despite the combination of different treatments, including surgery, radiotherapy, and chemotherapy, grade II and III meningiomas remain aggressive and are coupled with a poor prognosis and higher mortality [5]. Therefore, personalized therapy options that are more urgently required, providing improved therapy outcomes and ultimately, improve prognosis for patients with meningioma.

Weighted gene coexpression network analysis (WGCNA) is a powerful method to identify potential modules and biomarkers with correlation analyses between gene expression and clinical data [6]. The *WGCNA* package in R is able to implement the weighted coexpression network analysis in microarray datasets [6]. These analyses could identify significant prognostic biomarkers or therapeutic targets. This promising and reliable tool has been used to study numerous different diseases that have complex molecular mechanisms, such as colorectal cancer and glioblastoma multiforme [7, 8]. Additionally, there are two studies involving WGCNA of meningiomas five years ago. Based on the 3600 variable genes, Chang et al. identified that a module was associated with meningiomas using WGCNA and that four intramodular hub genes within that module (including GAB2, KLF2, ID1, and CTF1) were identified as oncogenic genes in other types of cancers [9]; however, the study lacked verification through further independent datasets. Another WGCNA study revealed a module named module Quantitative Trait Loci (mQTL), and single-nucleotide polymorphisms (SNPs) were significantly associated with meningioma stage, and the pathway analysis indicated that the hub genes in the module were involved in meningioma malignant conversion [10]. Though both studies identified the module related to the clinical information, there is a lack of verification for the reliability of these modules and hub genes, as well as preservation analyses.

In the present study, WGCNA was used to search for biologically meaningful modules based on two microarray datasets. The genes with high connectivity in the module were identified as hub genes. Moreover, validation analyses were implemented using two independent datasets. The results from the present study may contribute to revealing the pathogenesis of meningiomas and providing potential biomarkers for prognosis assessment and targeted therapy.

## 2. Material and Methods

### 2.1. Downloading and Preprocessing of Genetic and Clinical Data

Three microarray datasets including GSE43290, GSE74385, and GSE16581 were obtained from the Gene Expression Omnibus (GEO; http://www.ncbi.nlm.nih.gov/geo) database. GSE43290 (GPL96; Affymetrix Human Genome U133A Array) includes 47 meningioma samples and 4 normal samples with WHO grades [11]. GSE74385 (GPL10558; Illumina HumanHT-12 V4.0 expression beadchip) has 62 meningioma samples with WHO grades and prognosis status including recurrence and nonrecurrence [12]. Finally, GSE16581 (GPL570; Affymetrix Human Genome U133 Plus 2.0 Array) includes 68 meningioma samples with complete WHO grades [13]. Background correcting, normalizing, and log2 transformation were implemented to ensure clear data. Moreover, probes matching multiple genes were eliminated following annotations and were replaced by their average values. The top 5000 high standard deviation genes were selected for further analyses.

### 2.2. Construction of Weighted Gene Coexpression Network Analysis

The *WGCNA* package in R was implemented to construct gene coexpression modules related to WHO grades [6]. For achieving the standard of scale-free topology, an appropriate soft-thresholding value was analyzed when the scale independence power was set as 0.9 [14]. The acquired weighted adjacency matrix was then transformed into the topological overlap matrix (TOM). The smallest number was set as 30, and different modules were coded using different colors. The dissimilarity of module eigengenes (ME), the first principal component of the module, was calculated to evaluate the similarity of entire modules, and the modules were put together with a maximum cut-off value of 0.25 [15].

### 2.3. Module Preservation Analysis

Module preservation and quality statistics were calculated with the module preservation function (nPermutations = 200) to examine the stability of the acquired module using the *WGCNA* package [16]. The validation dataset GSE74385 contained the mRNA expression data of 62 samples. Those modules with high Zsummary and low medianRank scores were regarded as high conservative and stable modules, respectively.

### 2.4. Functional Annotation of Interested Module

The correlations between modules and clinical information such as WHO grades were computed by Pearson's correlation coefficient in order to select the biologically meaningful module. The gene significance (GS) and module membership (MM) were calculated in order to evaluate the clinical significance of the module. In addition, all genes of the modules were inputted into the Database for Annotation, Visualization and Integrated Discovery (DAVID; version 6.8; https://david.ncifcrf.gov) for functional analyses including Gene Ontology (GO) and Kyoto Encyclopedia of Genes and Genomes (KEGG) pathway analyses (*P* < 0.05) [17]. And Reactome pathway (https://www.reactome.org) analysis was conducted for the genes [18] and was visualized in Cytoscape (version 3.5.1; http://www.cytoscape.org) [19].

### 2.5. Identification of Hub Genes

According to the definition, the genes with high connectivity inside the module were regarded as hub genes. Based on the intramodular statistical analyses, the top 10 hub genes with high intramodular connectivity (IC) were selected for the subsequent analyses. Interaction networks of 10 hub genes were constructed using the Cytoscape software [19].

### 2.6. Efficacy Evaluations and Correlation Analyses

Efficacy evaluations and correlation analyses were performed to test the clinical value of the modules using three different datasets. An efficacy evaluation was presented with a receiver operator characteristic (ROC) curve using the *pROC* package in R [20]. When the area under the curve (AUC) value was greater than 0.7, the gene was considered to be capable of distinguishing meningioma samples from normal samples. The correlations between hub genes and WHO grades were presented in scatter plots, and their statistical significance was assessed using an independent sample *t*-test.

## 3. Results

### 3.1. Downloading and Preprocessing of Genetic and Clinical Data

**Figure 1:**
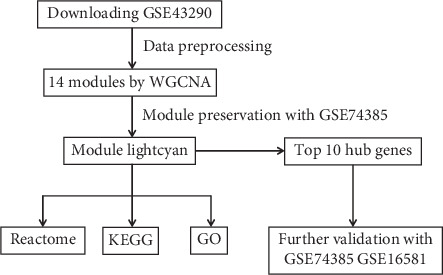
Flow chart presenting the design of present study. WGCNA: weighted gene coexpression network analysis; GO: Gene Ontology; KEGG: Kyoto Encyclopedia of Genes and Genomes.

A flow chart of the present study is presented in Figure 1. Three microarray datasets (GSE43290, GSE74385, and GSE16581) from the GEO database were obtained in the present study. Following preprocessing of the data, a total of 5,000 genes and 47 meningioma samples in the dataset GSE43290 were extracted for the next analyses.

### 3.2. Construction of Weighted Gene Coexpression Network Analysis

**Figure 2:**
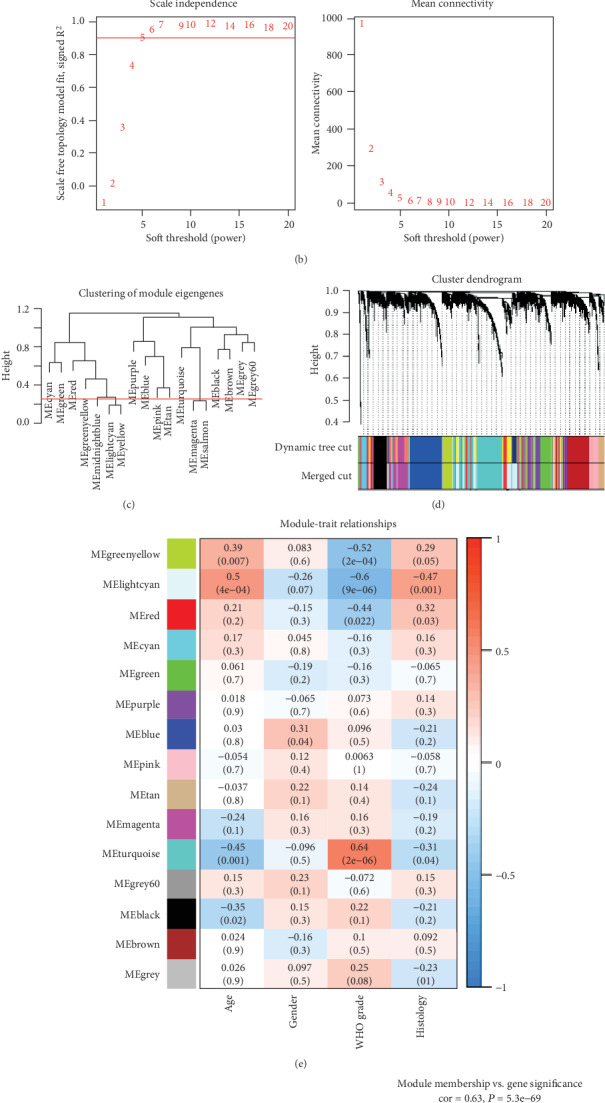
Construction of the weighted gene coexpression network analysis. (a) Cluster dendrogram of samples and the corresponding clinical traits. (b) Analyses of network topology for various soft-thresholding powers. (c) Clustering of module eigengenes was analyzed when modules were merged by a cut-off value of 0.25. (d) Clustering dendrograms of genes. (e) Module-trait relationships. (f) Gene significance of histology across modules. (g) Scatter plots for correlations between module membership and gene significance.

Following a quantity check, there was no sample outlier to be cleared (Figure 2(a)) and the soft-thresholding power was equal to 5 (Figure 2(b)). The weighed gene coexpression network was constructed based on the interaction patterns between genes (Figure 2). Gene coexpression modules, which included the clusters of genes with high topological overlap, were identified using average linkage hierarchical clustering and dynamic cut tree (Figure 2(d)). Genes that did not belong to any modules were allocated in the module grey. Following the clustering of ME, 14 coexpression modules were coded as different colors and the numbers of the each module ranged from 62 to 790 (Figure 2(d)). The 14 coexpression modules included black (286), blue (668), brown (539), cyan (115), green (375), greenyellow (197), grey60 (62), lightcyan (612), magenta (395), pink (243), purple (201), red (332), tan (182), and turquoise (790) (Figure 2(d)). In Figure 2(e), a heat map was showed to demonstrate the associations between coexpression modules and clinical information, and modules turquoise (*r* = 0.64, *P* = 2*e* − 06) and lightcyan (*r* = −0.6, *P* = 9*e* − 06) were the top 2 biologically meaningful among module-related WHO grades. In addition, the GS of each module is shown in Figure 2(f), and the value of module lightcyan was identified as the highest. The association between MM and GS of module lightcyan is shown in Figure 2(g), which further confirmed its biological significance.

### 3.3. Module Preservation Analyses

**Figure 3:**
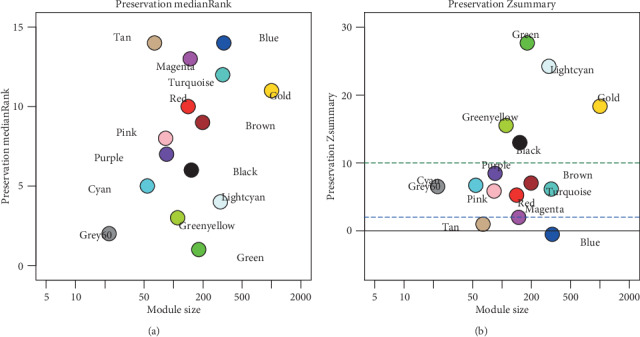
The medianRank and Zsummary values of the module preservation using the GSE74385 dataset. The medianRank graph (a) and the Zsummary graph (b). (b) The dashed blue and green lines indicate the thresholds Zsummary = 2 and Zsummary = 10, respectively. The fact that the medianRank trends to zero and Zsummary is greater than 10 demonstrated a higher degree of module preservation.

Through comparing the dataset GSE74385, which was regarded as the test cohort, the summary preservation statistics were analyzed and visualized. Modules green, lightcyan, and greenyellow, of which Zsummary statistics were greater than 10 and medianRank scores were the lowest, were revealed to be the most stable and preservative (Figure 3). By combining the biological significance-related grades and preservation, module lightcyan was selected and its genes were negatively related to WHO grades.

### 3.4. Functional Annotation of the Module of Interest

**Figure 4:**
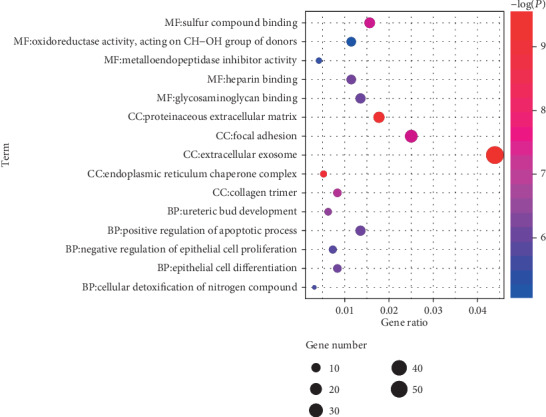
The plot for the top 5 GO enrichment annotations of all genes in module lightcyan. GO: Gene Ontology; BP: biological process; CC: cellular component; MF: molecular function.

**Figure 5:**
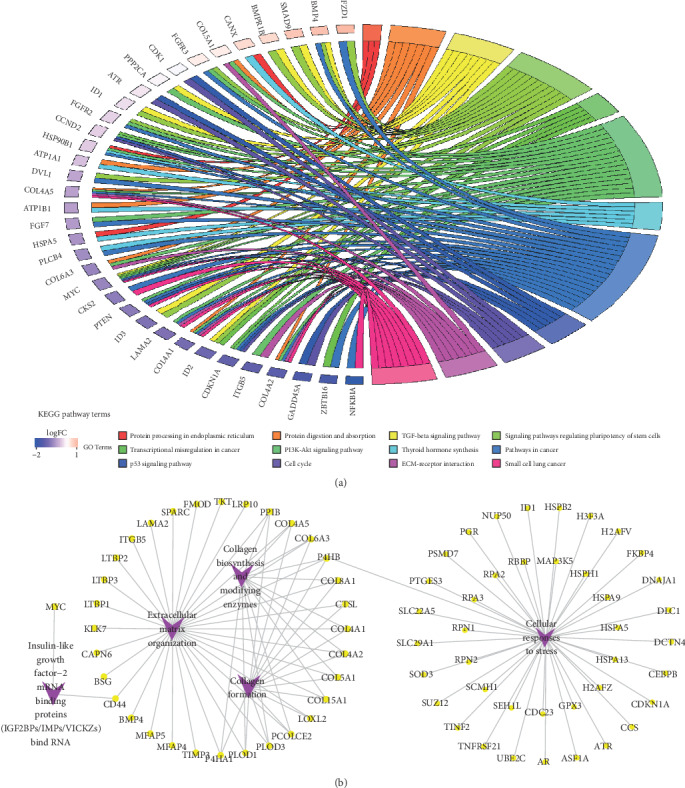
The plots for enriched KEGG and Reactome pathways of all genes in module lightcyan. (a) KEGG analysis was visualized using the package *GOplot* in R. The plot included 12 KEGG pathway terms. The genes with at least two pathway terms and the term which includes at least three genes are shown in the plot. (b) The top 5 enriched Reactome pathways were visualized in Cytoscape. KEGG: Kyoto Encyclopedia of Genes and Genomes.

A total of 612 genes in module lightcyan were used for the functional enrichment analyses. GO results showed that module lightcyan was primarily enriched in the negative regulation of epithelial cell proliferation, positive regulation of the apoptotic process, and epithelial cell differentiation in the biological process category; extracellular exosome in the molecular function category; and sulfur compound binding and heparin binding in the cell component category (*P* < 0.05; Figure 4). What is more, KEGG pathway results demonstrated that these genes were mostly enriched in the TGF-beta signaling pathway, cell cycle, and transcriptional misregulation in cancer (*P* < 0.05; Figure 5(a)). The Reactome pathway results showed that these genes were mostly associated with collagen formation, cellular responses to stress, and extracellular matrix organization (*P* < 0.05; Figure 5(b)). Thus, these findings indicated that the genes in module lightcyan played critical roles in the pathogenesis of meningiomas.

**Figure 6:**
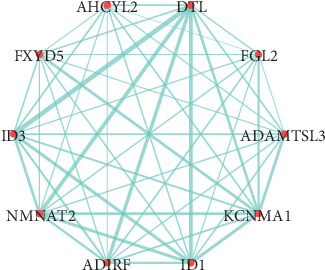
Visualization of the interaction network among the top 10 hub genes inside the module lightcyan in Cytoscape. The thickness of the line is positively associated with the degree of connectivity between two genes.

All of these findings may deserve a further study. With the aim of simplifying the area of research and promote its accuracy, the top 10 genes with high intramodular connectivity were subsequently extracted as hub genes in the study, which included DTL, ADAMTSL3, KCNMA1, ID1, ADIRF, NMNAT2, ID3, FXYD5, AHCYL2, and FGL2 (Figure 6).

### 3.5. Efficacy Evaluations and Correlation Analyses

**Figure 7:**
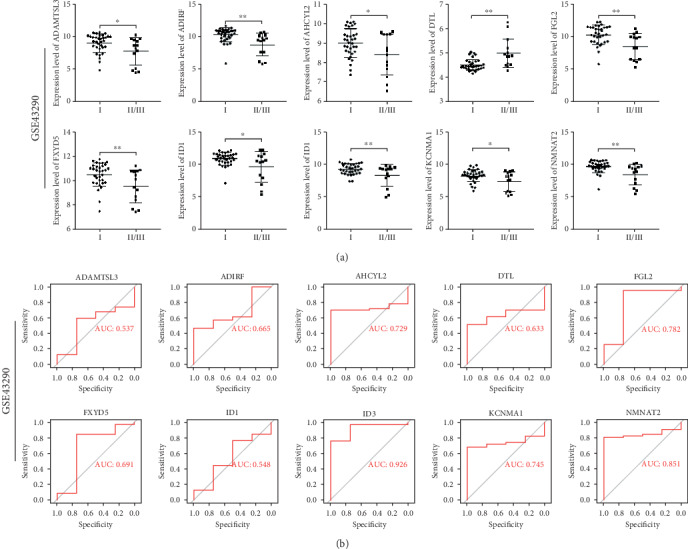
Correlation analyses and efficacy evaluations for hub genes in the GSE43290 dataset. (a) Scatter plots for hub genes of module brown across meningioma WHO grades. *P* values are the results of independent sample *t*-tests between grade I and grades II and III. (b) ROC analyses for the top 10 hub genes of module lightcyan. ROC curves and AUC statistics to evaluate the diagnostic efficiency of the hub genes distinguishing meningioma samples (*N* = 47) from normal samples (*N* = 4). WHO: World Health Organization; ROC: receiver operating characteristic; AUC: area under the curve.

Subsequently, ten hub genes (including DTL, ADAMTSL3, KCNMA1, ID1, ADIRF, NMNAT2, ID3, FXYD5, AHCYL2, and FGL2) were tested in the GSE43290 dataset and two independent cohorts, GSE74385 and GSE16581. In GSE43290, ten hub genes could distinguish between meningioma grades (*P* < 0.05; Figure 7(a)), which is consistent with the results of WGCNA. Though there were only four normal samples in GSE43290, the efficacy evaluation results still showed that five genes, including AHCYL2 (AUC = 0.729), FGL2 (AUC = 0.782), ID3 (AUC = 0.926), KCNMA1 (AUC = 0.745), and NMNAT2 (AUC = 0.851), could significantly distinguish meningiomas and normal samples (Figure 7(b)).

**Figure 8:**
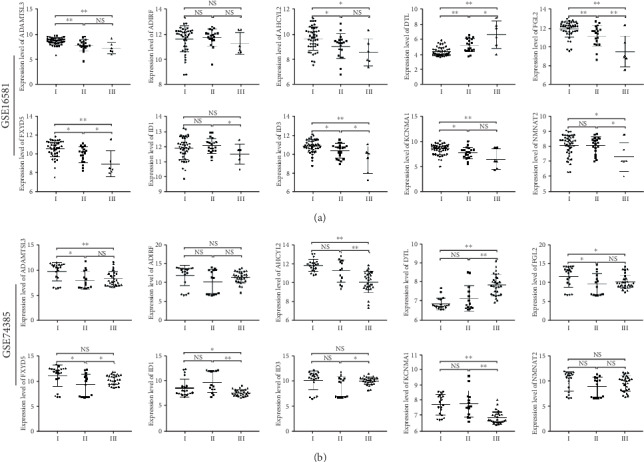
Scatter plots for hub genes of module brown across meningioma WHO grades in the GSE16581 and GSE74385 datasets. *P* values are the results of independent sample *t*-test between grade I and grades II and III in the (a) GSE16581 and (b) GSE74385 datasets. WHO: World Health Organization; NS: no significance; ^∗^*P* < 0.05; ^∗∗^*P* < 0.01.

**Figure 9:**
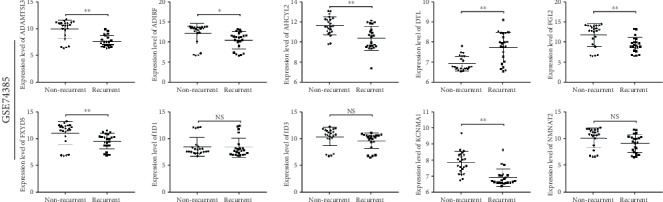
Scatter plots for hub genes of module brown between nonrecurrence forms and recurrence forms of meningiomas in the GSE74385 dataset. *P* values are the results of using independent sample *t*-tests between nonrecurrence samples and recurrence samples of meningiomas. NS: no significance; ^∗^*P* < 0.05; ^∗∗^*P* < 0.01.

The two independent datasets also showed that these genes were related to WHO grades (*P* < 0.05), except for ADIRF in GSE16581 and ADIRF and NMNAT2 in the GSE74385 dataset (*P* > 0.05), which confirmed them as potential biomarkers for predicting prognosis (Figure 8). What is more, these genes, excluding ID1, ID3, and NMNAT2, were confirmed to be able to differentiate between the recurrence and nonrecurrence forms of meningioma (*P* < 0.05; Figure 9).

## 4. Discussion

Meningiomas with a high recurrence rate are the most common primary intracranial tumor in adults and includes three grades [1]. Grade II and III meningiomas are aggressive, and the patients often tend to have a poor prognosis and high mortality [5]. Personalized therapy and management of meningioma are still lacking, for the underlying molecular mechanism remains unclear. Thus, further researches into the pathogenesis of meningioma are required. In the present study, WGCNA was performed to extract module lightcyan and the top 10 hub genes related to the WHO grades of meningioma.

WGCNA is a powerful systemic method to construct the coexpression networks, which has widely been implemented in a number of different types of diseases [21–23]. Compared with the two studies of WGCNA on meningiomas [9, 10], the present study highlighted the significant module associated with meningioma grade and the extracted hub genes were further confirmed by independent cohorts, which improved the reliability and objectiveness of the results. After an integrated bioinformatics analysis in the present study, module lightcyan including 612 genes was revealed to be significantly related to WHO grades (*r* = −0.6, *P* = 9*e* − 06). What is more, gene significance, module membership, and module preservation also confirmed the biological significance of module lightcyan.

A total of 612 genes inside module lightcyan were loaded the DAVID and Reactome pathway online tools for functional annotations. According to the GO analysis results, module lightcyan participates in cell apoptosis, proliferation, and differentiation, which play critical roles in tumor progression. Similar results were also observed in the KEGG and Reactome pathway analyses. For example, the activated PI3k-AKT signaling pathway is an important driver of tumor development in a number of different types of tumors, including meningioma [24, 25]. Members of the PI3K family are lipid kinases that participate in multiple cellular processes, such as differentiation, proliferation, and survival [25], which are related to the results of the GO analyses. Thus, the results of the present study further confirmed the biological significance of the module lightcyan.

A total of 10 hub genes, particularly AHCYL2, FGL2, and KCNMA1, were identified and confirmed by efficacy evaluations and correlation analyses based on three microarray datasets. Many research found that the top 10 hub genes were significantly associated with tumors. FGL2 has been identified as an important immune-suppressive modulator and as a potential immunotherapeutic target for treating gliomas [26]. A previous study found that silencing of FGL2 contributed to a significant decrease in cell viability and increase in cell apoptosis, accompanied decrease by ERK1/2, and p38 MAPK activation. Furthermore, it was revealed that overexpression of FGL2 is significantly associated with poor prognosis in patients with clear cell renal cell carcinoma [27]. These studies confirmed that FGL2 was involved in tumor progression and prognosis. KCNMA1 was demonstrated to be downregulated in grade III vs. grade I meningiomas using microarray expression profiles [28]. Based on the results of the Kaplan-Meier survival curve and experiments both *in vitro* and *in vivo*, KCNMA1 hypermethylation was significantly associated with shorter survival time in patients with GC (*P* = 0.036), which confirmed its prognostic value in gastric carcinogenesis [29]. PEST domain of AHCYL2 interacts with the NBCe1-B, which plays a critical role in neuronal modulation and intracellular pH regulation during activity [30, 31]. Reportedly, DTL overexpression decreased the protein level and accelerated the degradation rate of PDCD4 by ubiquitination and in cancer tissues was significantly upregulated than in normal tissues [32]. Moreover, cancer patients with higher DTL expression owned lower survival rate, and functional experiments found that DTL enhanced the motility and proliferation of cancer cells through degrading PDCD4 to promote the development of cancers [32]. A recent study showed that DTL, as one of the nine hub genes, played a role in type 2 diabetes mellitus and hepatocellular carcinoma (HCC) [33]. Genome-wide CRISPR Knockout Screens identified that ADAMTSL3 and PTEN were significantly related to HCC proliferation and metastasis and DAMTSL3 and PTEN were downregulated in HCC cells than in normal liver cells [34]. And HCC patients with low expression had a poor survival time, and further biological experiments *in vitro* and *in vivo* confirmed that DAMTSL3 and PTEN promoted the proliferation and metastasis of HCC cells [34]. A recent study reported that ID1 acted as a transcriptional regulator and played a critical role for glioblastoma initiation and chemoresistance, and ID1 knockdown promoted the treatment effect of temozolomide, delays tumor recurrence, and prolongs survival [35]. Reportedly, a recent study found that mitochondrial impairment activated the Wallerian pathway and caused the axon degeneration by the depletion of NMNAT2 [36]. Moreover, ID3 was reported to act as a biomarker promoting the stemness of intrahepatic cholangiocarcinoma by promoting the transcriptional activity of *β*-catenin [37]. Furthermore, high-grade serous ovarian carcinoma patients with higher expression of FXYD5 owned a shorter survival time than patients with lower expression of FXYD5, and FXYD5 played a critical role in survival and prognosis of HCSOC [38]. Similarly, biological experiments *in vitro* and *in vivo* showed that FXYD5 promoted the metastasis of ovarian cancer cells via TGF-*β*/SMAD signaling pathways [39]. Besides, a study demonstrated that FXYD5/Dys could serve as a biomarker of endometrial cancer progression associated with TGF-*β*1 and NF-*κ*B signaling pathways [40]. The findings from the present study confirmed the reliability and accuracy of hub genes as prognostic biomarkers in tumors, particularly in meningiomas.

However, the present study has some limitations. The low sample size may mean that the results are not as accurate as they could be. There were only 4 normal samples in the GSE43290 dataset and only 6 grade III meningioma samples in the GSE16581 dataset, which affected the analyses. Larger samples are required in future studies. Despite the small sample size, the plots still demonstrate relevant trends to a certain extent. Therefore, further functional research needs to be done to confirm the roles of the top 10 hub genes in meningioma pathogenesis in the future.

## 5. Conclusion

A total of 14 coexpression modules of meningiomas were constructed and identified using WGCNA. The integrated bioinformatics methods confirmed the stability and preservation of module lightcyan-related WHO grades. The GO enrichment, KEGG, and Reactome pathway analyses confirmed that the 612 genes of the module were significantly associated with meningioma progression. Moreover, the top 10 hub genes were selected based on intramodular connectivity and further confirmed to be associated with the grade and prognosis of meningiomas when using an independent sample *t*-test and ROC curve in three datasets. The results of the present study not only provide coexpression modules for an improved understanding of its pathogenesis but also provide potential biomarkers that associated with tumor grades and recurrence, allowing the prediction of prognosis and progression in patients with meningiomas.

## Data Availability

The data used to support the findings of this study are available from the corresponding author upon request.
